# Heteroresistance to amikacin in *Klebsiella aerogenes* isolates from patients in an intensive care unit in Brazil

**DOI:** 10.1128/spectrum.02833-25

**Published:** 2026-02-26

**Authors:** Natália Ribeiro Crispim, Gustavo Dantas Nunes, Gabriela Guerrera Soares, Rebecca Elizabeth Shilling, Roumayne Lopes Ferreira, Leslie Camelo Campos, Olinda Soares Athaide Alcobaça, João Pedro Maia de Oliveira da Silva, Saulo Henrique Rodrigues, André Pitondo-Silva, Andrea Soares da Costa Fuentes, Anderson Ferreira da Cunha, Robert J. Mitchell, Maria-Cristina da Silva Pranchevicius

**Affiliations:** 1 Departamento de Genética e Evolução, Universidade Federal de São Carlos 67828, São Carlos, São Paulo, Brazil; 2 Laboratório Central de Saúde Pública do Tocantins, Palmas, Tocantins, Brazil; 3 Programas de Pós-graduação em Odontologia, Biotecnologia e Tecnologia Ambiental, Universidade de Ribeirão Preto, Ribeirao Preto, Brazil; 4 Department of Biological Sciences, Ulsan National Institute of Science and Technology, Ulsan, South Korea; Health Canada, Ottawa, Canada

**Keywords:** *Klebsiella aerogenes*, heteroresistance, amikacin, whole-genome sequencing, resistance genes, intensive care unit

## Abstract

Heteroresistance is defined by the presence of subpopulations within a bacterial isolate that exhibit greater antibiotic resistance than the dominant population. In this study, we investigated amikacin heteroresistance in the opportunistic, nosocomial pathogen *Klebsiella aerogenes* (AHR-KA). Eight carbapenemase-resistant, but amikacin-susceptible, isolates from intensive care units of a Brazilian hospital were analyzed. Population analysis profiling identified five amikacin heteroresistant isolates (AHR-KA-1 to 5), with subpopulation frequencies ranging from 1.83 × 10^−7^ to 6.01 × 10^−6^. Among these, only AHR-KA-1 exhibited stable heteroresistance following serial passaging in antibiotic-free media. AHR-KA-1 demonstrated only slightly reduced growth rates when compared with those of the unstable AHR-KAs, parental, and control strains, suggesting no significant fitness cost associated with drug resistance. Time-kill assays for AHR-KA-1 showed an initial decline in cell viability, followed by regrowth at both 4× and 8× MIC. The draft genome of the stable isolate had a total length and G+C content similar to most of the sequenced genomes from *K. aerogenes*. Notably, AHR-KA-1′s *aac(6*′*)-Ib-cr* variant contained D179Y and R102W mutations, conferring amikacin resistance. Moreover, quantitative reverse transcription PCR revealed significantly elevated expression of the *aac(6*′*)-Ib-cr* gene in AHR-KA-1 before amikacin-free passage, compared to both the parental strain and the AHR-KA-1 strain after drug-free passage. Therefore, this study identified amikacin heteroresistance in carbapenemase-producing *K. aerogenes*, including a stable, mutation-driven phenotype with low fitness cost and rapid regrowth under high amikacin concentrations, underscoring its clinical relevance. These findings reinforce the importance of detecting heteroresistant subpopulations and strengthening surveillance to prevent treatment failure and the spread of undetected resistance.

**IMPORTANCE:**

*Klebsiella aerogenes*, an opportunistic human pathogen, is frequently implicated in severe and invasive infections, particularly in immunocompromised individuals. The growing prevalence of antibiotic resistance among these strains poses a significant therapeutic challenge. The phenomenon of heteroresistance further complicates management, potentially leading to diagnostic difficulties due to the lack of standardized detection methods and subsequent treatment failures. Our studies identified and characterized *K. aerogenes* strains heteroresistant to amikacin, isolated from patients in an intensive care unit. Such data can serve as a foundational reference for understanding the clinical relevance, genomic variability, and pathogenic potential of *K. aerogenes* heteroresistance to antibiotics used in clinical settings.

## INTRODUCTION

*Klebsiella aerogenes*, formerly known as *Enterobacter aerogenes*, is an opportunistic Gram-negative bacterium responsible for a wide range of infections, including meningitis, septicemia, and wound, urinary, and respiratory tract infections (1). It is also considered an important emerging multidrug-resistant (MDR) pathogen as it is associated with a high mortality rate among patients in intensive care units (2, 3).

Carbapenems are a class of β-lactam antibiotics widely utilized to treat infections caused by multidrug-resistant Gram-negative bacteria. These antibiotics exhibit broad-spectrum antibacterial properties by inhibiting transpeptidases, which prevents peptidoglycan synthesis and ultimately results in bacterial cell lysis (4). However, the growing use of carbapenem antibiotics has contributed to the emergence of carbapenem-resistant *Enterobacteriaceae* strains, such as *K. aerogenes* (CRKA). These new strains pose a growing challenge in clinical settings, owing to their rapid spread and limited treatment options (5). While polymyxins and tigecycline have traditionally been viewed as preferred therapeutic options for infections involving carbapenem-resistant *Enterobacteriaceae*, emerging resistance to these antimicrobial agents has also become a growing concern (6).

In clinical settings, Gram-negative pathogens that are highly resistant to other antimicrobials, such as carbapenems, maintain susceptibility to aminoglycoside antibiotics, including amikacin, gentamicin, tobramycin, and plazomicin (7). Among these, amikacin is considered the empirical treatment choice for nosocomial infections or settings with a high prevalence of extended-spectrum β-lactamase-producing strains (8). It is frequently used in mono- or combination regimens to manage severe infections (9), and its importance lies in its efficacy as it is less susceptible to the actions of aminoglycoside-inactivating enzymes (10). However, the emergence of amikacin heteroresistance in *K. aerogenes* strains may compromise its clinical efficacy, highlighting the need for continued monitoring and research into this phenomenon (11, 12).

Heteroresistance refers to a phenomenon in which a subpopulation within a susceptible microbial population exhibits antimicrobial resistance. Following antibiotic exposure, these resistant subpopulations may increase in frequency, potentially leading to treatment failure (13–15). Heteroresistance was first reported in a *Haemophilus influenzae* subpopulation with increased streptomycin resistance (16) and can be classified as either mono- or polyclonal, where monoclonal heteroresistance arises from heterogeneity within a single clonal population, while polyclonal heteroresistance arises from populations with different genotypes exhibiting varying levels of resistance. Among these, monoclonal heteroresistance is often unstable, and the resistant cell line becomes susceptible once the antibiotic pressure is removed (17).

In recent years, heteroresistance has attracted significant attention and has been investigated across a range of bacteria, including Gram-negative strains when exposed to colistin, tigecycline, ampicillin-sulbactam, amikacin, carbapenems, or cefiderocol (18–21). The underlying genetic mechanisms of heteroresistance remain poorly understood, however (18). In this study, therefore, we conducted a detailed analysis of amikacin heteroresistance in *K. aerogenes* clinical isolates from Brazil.

## MATERIALS AND METHODS

### Bacterial isolates

This study initially screened eight CRKA isolates for amikacin heteroresistance. These isolates were obtained from clinical samples and medical devices in the neonatal and adult intensive care units of a tertiary public hospital in Palmas, Tocantins, Brazil. These strains were characterized phenotypically and genotypically in a prior study (22). All exhibited carbapenemase resistance but remained susceptible to amikacin, as determined using the VITEK-2 compact automated system (bioMerieux, France) and the Kirby–Bauer disk diffusion method (22).

### Selection of amikacin heteroresistant candidate isolates

A disk-diffusion assay was used to screen for amikacin heteroresistant isolates. For this, *K. aerogenes* cultures grown in tryptic soy broth (TSB) (Merck, Germany) were adjusted to a 0.5 McFarland standard in a saline solution (0.9% NaCl) and inoculated onto Mueller-Hinton agar (MHA) (Merck, Germany). Disks containing 30 mg of amikacin (DME, Brazil) were placed on the agar, and the plates were incubated at 37°C for 24 h. Any colonies that grew within the zone of inhibition were considered potential amikacin heteroresistant *K. aerogenes* (AHR-KA) strains. The amikacin zone diameter breakpoints for *K. aerogenes* were interpreted using the European Committee on Antimicrobial Susceptibility Testing (EUCAST, 2024), where a susceptible breakpoint is defined as values ≥18 mm.

### Population analysis profiling and frequency analysis

To confirm the heteroresistance phenotype, population analysis profiling (PAP) was conducted as previously described by Zhang et al. (11) with some modifications. To determine heteroresistance, a 20 μL aliquot of the AHR-KA strain in TSB (Merck, Germany) was inoculated into 2 mL of fresh TSB (Merck, Germany) and incubated overnight at 37°C. After overnight incubation, the cultures were diluted in a saline solution to 0.5 McFarland. Ten-fold serial dilutions were prepared, and 10 μL aliquots were plated onto amikacin-containing (0.5 to 128 mg/L) tryptic soy agar (TSA) (Merck, Germany) plates. In parallel, 10 μL aliquots were also plated on drug-free cation-adjusted MHA (CA-MHA) (Merck, Germany) plates. After growth overnight, the viable counts on each were used to calculate the frequency of heteroresistant isolates as described previously (23). Heteroresistance was defined by the presence of isolates at a frequency greater than 10^−7^ and with a resistance level that exceeded the breakpoint by at least eightfold (24). *Klebsiella pneumoniae* ATCC 13883 (American Type Culture Collection—ATCC, USA) was used as a control bacterium, as it is widely used in research and diagnostics. All of these tests were performed in duplicate.

### Passage stability

Colonies from each PAP plate containing amikacin at concentrations of 64 mg/L were inoculated into fresh TSB (Merck, Germany) and incubated overnight at 37°C. The resulting cultures were then streaked onto TSA (Merck, Germany) plates containing amikacin at the same concentration of 64 mg/L. Following overnight growth at 37°C, individual colonies from each plate were transferred to fresh cation-adjusted Mueller-Hinton broth (MHB) (Merck, Germany), and their amikacin minimum inhibitory concentrations (MIC) were determined. To evaluate the stability of the heteroresistance phenotypes, cultures grown on fresh MHB (Merck, Germany) at an amikacin concentration of 64 mg/L (previously analyzed using TSA [Merck, Germany] plates containing amikacin and MICs) were serially passaged in MHB (Merck, Germany) without antibiotics at a 1,000-fold dilution daily for 10 days, which is approximately 100 generations (25). After the final passage, the amikacin MICs were determined once more using the broth microdilution method (26). The heteroresistance phenotype was considered unstable if the MIC decreased or returned to that of the original parental isolate in at least one of the cultures (27, 28). The amikacin breakpoints are based on the guidelines set by the European Committee on Antimicrobial Susceptibility Testing, where a value of ≤8.0 mg/L was deemed susceptible, and a value of >8.0 mg/L resistant. *K. pneumoniae* ATCC 13883 (ATCC, USA) was once more used as a control.

### Growth curves

Growth kinetics of each strain were investigated in the absence of antibiotics and were conducted as previously described by Jayol et al. (29), with some modifications. Specifically, 60 mL lysogeny broth (LB) (Merck, Germany) cultures were inoculated to an initial density of 10^8^ CFU/mL and incubated at 37°C. The optical densities at 600 nm (ODs) were measured hourly for 12 h using a spectrophotometer (KASVI, Brazil), and a growth curve was generated based on the average OD values from three independent cultures for each strain. The Gompertz model was subsequently used to predict the moment of stabilization and growth plateau for each (30).

### Time-kill assays

Time-kill assays were conducted using the stable AHR-KA-1 as described by Perim et al. (31), with some modifications. Tubes containing 10 mL of freshly prepared cation-adjusted MHB (HiMedia, India) were inoculated with AHR-KA-1 to an initial density of approximately 5 × 10^5^ CFU/mL. Amikacin was then added to achieve final concentrations of 0, 4, 8, 16, 32, 64, or 128 mg/L, and the cultures were shaken at 150 rpm and maintained at 37°C. Samples were collected at 0, 4, 8, 12, and 24 h. These samples were then used to prepare a 10-fold dilution series, ranging from 10^1^ to 10^8^. Aliquots from these dilutions were spotted onto antibiotic-free MHA (Merck, Germany) plates to determine the total cell population and assess the frequency of resistant isolates. These plates were incubated at 37°C for 24 h, and their CFU/mL were calculated. Bactericidal activity was defined as a ≥3 log_10_ CFU/mL reduction in the total CFU/mL from the original inoculum (32). All experiments were performed in triplicate.

### Biofilm formation

These experiments were conducted in quadruplicate, using 96-well flat-bottom plates, following the methods previously described by Stepanović et al. (33). Each well was filled with 180 μL of LB (Merck, Germany) along with 20 μL of bacterial suspension cultured in LB and adjusted to a 0.5 McFarland standard. The plate was then incubated at 37°C for 48 h. After this period, the medium was removed without disturbing the biofilm, and the wells were gently washed with phosphate-buffered saline (HiMedia, India) to remove non-adherent bacteria. The biofilms were then fixed with 95% ethanol for 15 min and allowed to dry. Subsequently, the plates were stained with 100 μL of 1% crystal violet (Dinâmica, Brazil) solution for 15 min. The excess dye was removed by washing the biofilms with sterile distilled water, and the plates were air-dried. The biofilm biomass was then quantified by resolubilizing the crystal violet in 200 μL of 30% acetic acid for 10 min and measuring the OD of the sample at 540 nm in a microplate reader (KASVI, Brazil). The OD values for each isolate were obtained by averaging the values from four independent tests. The OD cutoff (ODc) was defined as three standard deviations above the mean OD of the negative control. The isolates were classified into four categories based on the average optical densities in relation to the results obtained for the ODc: non-adherent if OD ≤ ODc; weakly adherent if ODc < OD ≤ 2 × ODc; moderately adherent if 2 × ODc < OD ≤ 4 × ODc; or strongly adherent if 4 × ODc < OD.

### Biochemical identification

CRKA532 and all AHR-KA isolates were identified using Bactray 1, 2, and 3 systems (Laborclin, Brazil) following the manufacturer’s protocol. Single colonies grown overnight at 37°C on LB agar were suspended in the supplied diluent to a 0.5 McFarland standard. Aliquots were inoculated into all reaction wells of the appropriate Bactray tray, which was then incubated aerobically at 37°C for 18 h. Reactions were scored as positive or negative using the manufacturer’s color chart, and the resulting biochemical profiles were compared with the corresponding Bactray 1, 2, or 3 identification tables to determine the most probable bacterial species.

### RNA isolation and transcriptional analysis using quantitative RT-PCR

Cultures of the parental CRKA and AHR-KA-1 were grown in TSB (Merck, Germany) medium at 37°C with shaking until mid-log phase (OD 0.6), at which point the bacterial cells were pelleted by centrifugation (8,000 × *g*, 5 min) and the RNA was extracted using 1 mL Trizol (Invitrogen, USA) according to the manufacturer’s protocol. The quality and concentration of each sample were assessed by denaturing agarose (Uniscience, USA) gel (2.2 M formaldehyde; 1.2% [wt/vol] agarose) electrophoresis and a nanophotometer (NanoVue, GE HealthCare, USA), respectively. Five micrograms of total RNA were treated with Turbo DNase I (Invitrogen, USA) to remove any genomic DNA, and the RNA was reverse transcribed with the High-Capacity cDNA Reverse Transcription kit (Life Technologies, USA) using random primers. Real-time RT-PCR was conducted in triplicate using the GoTaq qPCR Master Mix (Promega, USA) with three independent biological samples in a QuantStudio 6-Flex Real Time PCR System (Applied Biosystems, USA). The primers used in these tests were selected based on published criteria (34), while non-template controls were used to confirm the absence of contaminating DNA in every run. The 16S rRNA gene was employed as an internal control to normalize the expression of each candidate gene. The threshold cycle numbers were determined by the detection system software, and the data were analyzed using the 2^−ΔΔCt^ method (28). Quantitative real-time PCR (qRT-PCR) was used to measure the expression of the *aac(6′)-Ib-cr*, *acrD*, *cpxA*, and *emrE* genes, utilizing specific oligonucleotide primers (Table S1), designed using the Integrated DNA Technology program (https://www.idtdna.com/scitools/Applications/RealTimePCR/default.aspx). All these experiments were conducted in triplicate with three independent RNA samples.

### DNA isolation

Genomic DNA from each *K. aerogenes* isolate was extracted from overnight cultures using the Cellco Genomic DNA purification kit (Cellco Biotech, Brazil), following the manufacturer’s instructions. The DNA purity was measured using a NanoVue Plus (GE Healthcare, United States), quality through electrophoresis, and concentration using a Qubit 3.0 fluorometer in combination with the Qubit dsDNA Broad Range Assay Kit from Life Technologies (Carlsbad, CA, USA).

### Detection of antibiotic-resistant genes

PCR was conducted to identify resistance-related genes, including those encoding carbapenemases, extended-spectrum beta-lactamases, aminoglycoside-modifying enzymes, and efflux pumps, within the heteroresistant isolates before and after the passage stability assay. Table S1 provides details on the amplicon lengths and PCR parameters employed.

### Genome sequencing of AHR-KA 1

A Nextera DNA Sample Prep Kit (Illumina, USA) was used to prepare a 2 × 150 bp paired-end library for whole-genome sequencing on an Illumina NextSeq 2000 platform (Illumina, USA). The library’s quality and DNA fragment size were evaluated using electrophoresis with a 1.5% agarose gel and quantified using a fluorometric method involving the Qubit 3.0 instrument and the Qubit dsDNA Broad Range Assay Kit (Life Technologies, USA).

### Genome assembly and annotation

Quality control of the raw reads was assessed using the FastQC program v.0.12.1 (http://www.bioinformatics.babraham.ac.uk/projects/fastqc/). Sequencing data generated by NextSeq 2000 was processed in the BaseSpace cloud by the BCL Convert program (Illumina, USA), which converted raw BCL-format data into FASTQ files, associating each base with a Phred quality score (35). Genome assembly was conducted using the Shovill program v.1.1.0 (https://github.com/tseemann/shovill), with default parameters plus “--minlen 500 --mincov 30 --depth 250.” Plasmid detection was performed using PlasmidFinder 2.13 (https://cge.food.dtu.dk/services/PlasmidFinder/) (36).

The genome map was generated using Proksee (https://proksee.ca/) (37) while annotation was performed using Bakta version 1.9.3 (38) and the server Rapid Annotations using Subsystems Technology (https://rast.nmpdr.org/) (39). The completeness of the assembled genome was evaluated with BUSCO v.5.7.1 (40).

### Characterization of the resistome

Genes associated with antibiotic resistance, efflux pumps, and porins were annotated using the following online tools with their default settings: Comprehensive Antibiotic Resistance Database (CARD) (https://card.mcmaster.ca/analyze/rgi) (41), ResFinder v.4.6.0 (http://genepi.food.dtu.dk/resfinder) (42), and BlastKOALA (https://www.kegg.jp/blastkoala/) (43). Additionally, BLAST searches were performed against CARD (41) and Antibiotic Resistance Gene-ANNOTation (ARG-ANNOT) (https://www.mediterranee-infection.com/acces-ressources/base-de-donnees/arg-annot-2/) (44). Database search parameters were set to an e-value ≤1E^−10^, identity ≥70%, and coverage ≥90%.

### Statistical analysis

Statistical analysis using Student’s *t*-test was performed with GraphPad Prism 7.0 software to analyze the continuous data from biofilm formation. Gene expression analysis by RT-qPCR was performed using the Kruskal-Wallis test, which is appropriate for non-parametric data. This test was used to compare all samples. Differences were considered statistically significant when the *P* value was less than 0.05.

## RESULTS

### Detection of amikacin-resistant subpopulations in CRKA and a comparison of the growth rates of these resistant subpopulations

**Fig 1:**
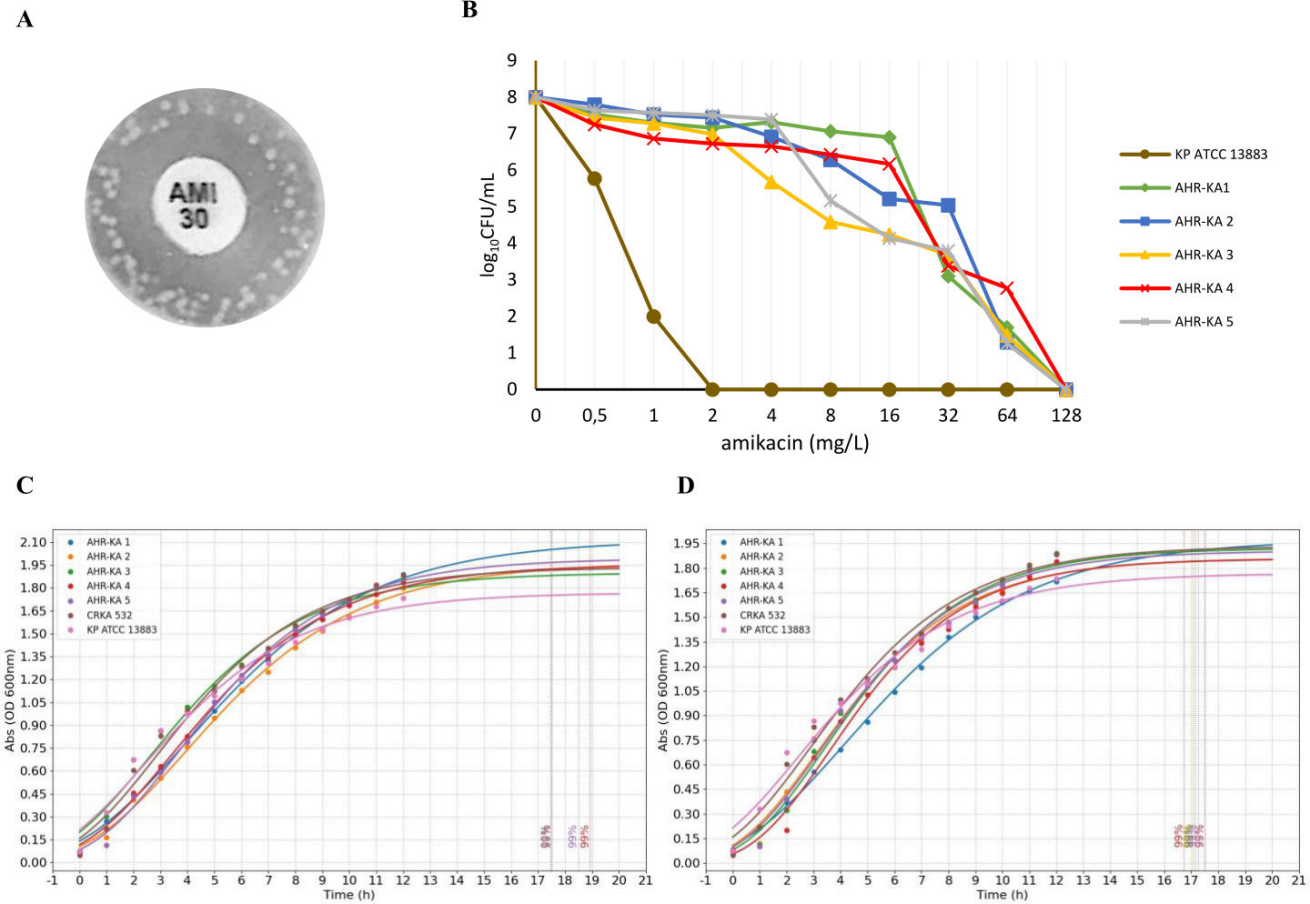
Amikacin heteroresistance in a carbapenem-resistant *K. aerogenes* strain. (**A**) The amikacin heteroresistance of the carbapenem-resistant *K. aerogenes* isolates was detected using the disk diffusion method. (**B**) PAP of the amikacin-heteroresistant *K. aerogenes* isolates and the control strain *K. pneumoniae* ATCC 13883. The x-axis shows the amikacin concentrations (µg/mL) used to select subpopulations exhibiting higher levels of resistance, while the y-axis represents the frequency of bacterial cells expressed as log₁₀ CFU/mL. Growth rates were compared among the AHR-KA heteroresistant strains, the parental strain, and the *K. pneumoniae* ATCC13883 reference strain before (**C**) and after (**D**) drug-free passaging.

In this study, we utilized eight CRKA that our research group had previously characterized as amikacin-susceptible using the VITEK2 compact automated system (22) to identify amikacin heteroresistant isolates. One CRKA532 isolate, with a MIC of 4 mg/L and a zone diameter greater than 18 mm, displayed distinct colonies within the inhibition zone in the disc diffusion assay (Fig. 1A), suggesting the presence of amikacin heteroresistant subpopulations. Five of these colonies (AHR-KA-1 to AHR-KA-5) were subjected to PAP (Fig. 1B). All five isolates grew on plates containing 64 mg/L amikacin, representing a 16-fold increase in the MIC when compared to their parental strain (MIC = 4 mg/L). Moreover, another related strain, *K. pneumoniae* ATCC 13883, was unable to grow at concentrations >2 µg/mL (Fig. 1B). The heteroresistant isolate frequency ranged from 1.83 × 10^−7^ to 6.01 × 10^−6^, and all except one isolate were unstable (Table 1). The fitness costs of amikacin-heteroresistance before and after a drug-free passage resulted in only slightly reduced growth rates (Fig. 1C and D), suggesting this phenotypic shift did not significantly impair bacterial fitness.

**TABLE 1:** PAP demonstrating amikacin heteroresistance in five clinical *K. aerogenes isolates*^*a*^

Isolates	Highest concentration in PAP where growth occurred (mg/L)	PAP frequency	MIC of isolates after PAP (mg/L)	MIC of isolates grown for 10 days without amikacin (mg/L)	Heterogeneous phenotype
AHR-KA-1	64	5.00 × 10^−7^	64	64	Stable
AHR-KA-2	64	1.94 × 10^−7^	128	8	Unstable
AHR-KA-3	64	3.04 × 10^−7^	128	4	Unstable
AHR-KA-4	64	6.01 × 10^−6^	64	32	Unstable
AHR-KA-5	64	1.83 × 10^−7^	128	64	Unstable
*K. pneumoniae* ATCC 13883	≤ 2	9.9 × 10^−7^	≤2	NA	NA

^*a*^ MIC, minimum inhibitory concentration. NA, not analyzed. The amikacin MICs and zone diameter breakpoints for *K. aerogenes* were interpreted using the European Committee on Antimicrobial Susceptibility Testing (EUCAST, 2024) susceptible breakpoint, with values of ≤8 mg/L and ≥18 mm, respectively. A *K. pneumoniae* ATCC13883 strain was used as the control.

### Biochemical characterization

Basic biochemical profiling of all five AHR-KA isolates, both in the presence and absence of antibiotics, was conducted using 24 biochemical tests, with results provided in Table S2. All isolates exhibited identical biochemical profiles, suggesting a common origin for the selected colonies. In addition, the AHR-KA isolates displayed a positive ornithine decarboxylase (ODC) reaction and an absence of malonate utilization, biochemical traits associated with *K. aerogenes* and commonly used for its initial phenotypic identification (45–47).

### Resistance genes and biofilm formation

**Fig 2:**
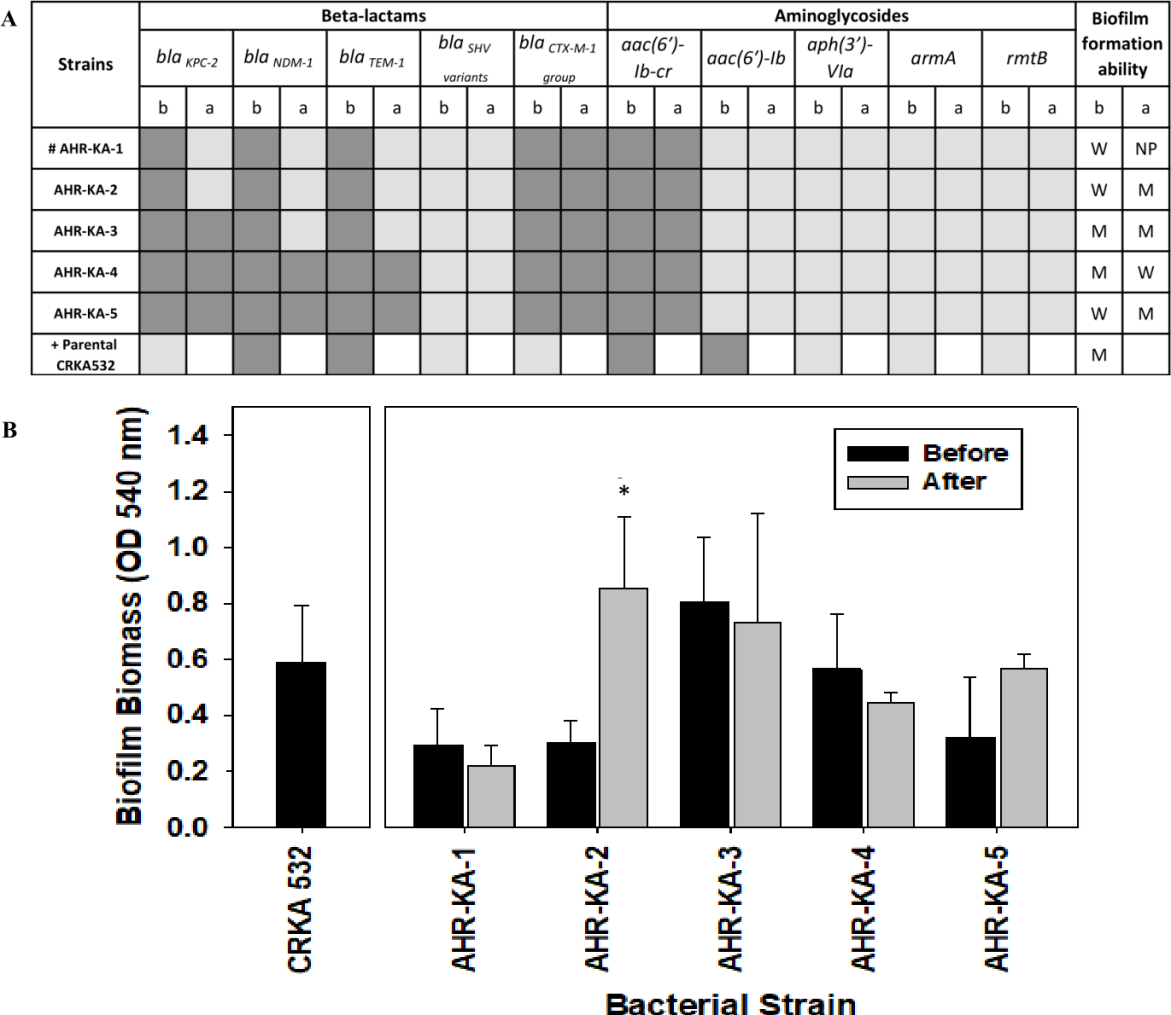
Characterization of antibiotic resistance genes and biofilm formation in the amikacin-heteroresistant *K. aerogenes* isolates. (**A**) Genes coding for resistance and efflux pumps before (**B**) and after (**A**) passaging in the absence of the amikacin antibiotic for 10 days. **+** Genes associated with drug resistance in the amikacin-susceptible parental CRKA532 strain were analyzed as described by Rodrigues et al. (22). Square: white (not checked), light gray (lost gene), dark gray (present gene). # Whole-genome sequencing was performed on AHR-KA-1. (**B**) Comparison of biofilm formation between samples collected before and after amikacin removal. W: weak biofilm producer; M: moderate biofilm producer; NP: non-producer. The amikacin-susceptible CRKA532 parental strain was used as the control. *Statistically significant (*t*-test: *P* < 0.05).

Our group previously identified the genes responsible for drug resistance in the parental strain (CRKA532) (22). Consequently, the stability of the various β-lactam and aminoglycoside resistance genes in all five amikacin-heteroresistant isolates was assessed, but the parental strain was not subjected to drug-free passaging due to its amikacin susceptibility (white square in Fig. 2A). Prior to drug-free passaging, all five AHR-KA isolates carried the *bla*_NDM-1_ and *bla*_KPC-2_ genes (Fig. 2A) (dark gray) but, in the absence of selective pressure, two strains (AHR-KA-1 and AHR-KA-2) lost both genes and one strain (AHR-KA-3) lost only the *bla*_NDM-1_ gene (light gray). Similar results were seen with the *bla*_TEM_ gene, while the *bla*_CTX-M-1_ gene group, which confers resistance to β-lactams, such as penicillins and cephalosporins, was stably present in all five strains. Focusing on aminoglycoside resistance markers, only the *aac(6')-Ib-cr* gene was detected in the AHR-KA isolates (dark gray), and this gene was stable (Fig. 2A).

Biofilm formation by the heteroresistant *K. aerogenes* isolates was also assessed both before and after drug-free passaging. One unstable strain (AHR-KA-2) formed more robust biofilms after undergoing drug-free passaging, suggesting heteroresistance in this isolate may suppress biofilm formation. The stable heteroresistant strain (AHR-KA-1), however, did not show improvement in biofilm formation after passaging (Fig. 2B). As such, the potential relationship between these two phenotypes merits further downstream study.

### Time-kill assay for amikacin monotherapy

**Fig 3:**
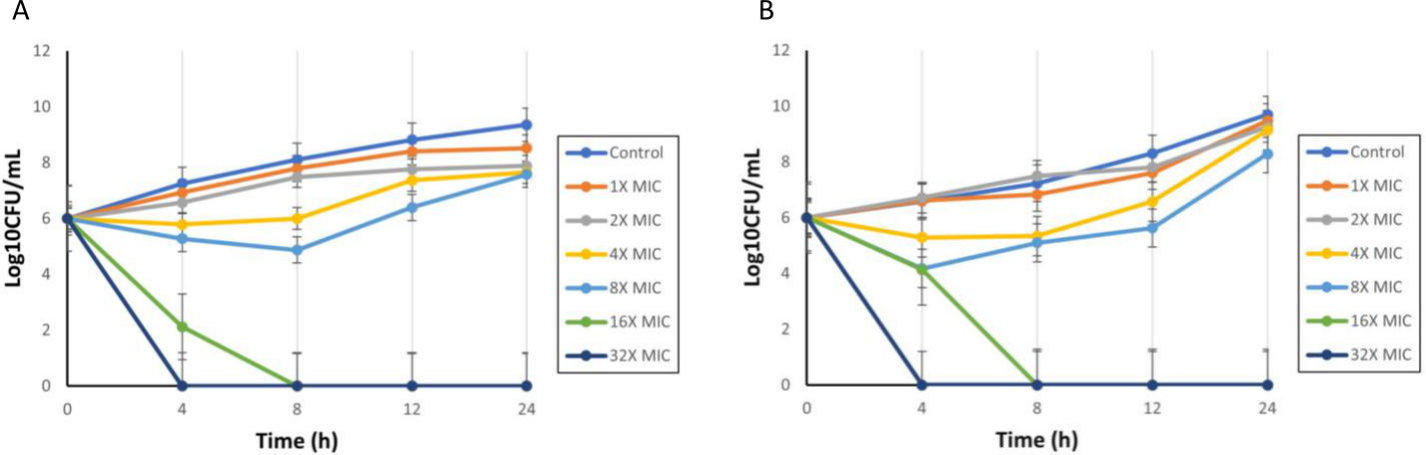
Time-kill assays. The growth of the stable AHR-KA-1 strain was examined both before (**A**) and after (**B**) the removal of the amikacin antibiotic. The MHA plates without amikacin were used as controls.

Heteroresistance, unlike persistence, is a type of antibiotic resistance where bacterial growth initially decreases, then increases in time-kill curve assays since a small subpopulation of cells survives the exposure and multiplies (48). This was explored with our stable isolate (AHR-KA-1) using cultures both before and after drug-free passaging (Fig. 3A and B). For both, the results were basically identical, with initial drops in viability when the antibiotic concentration was 8× MIC, followed by regrowth, while higher concentrations led to the complete killing of the cultures. Taken together with the PAP assay results in Table 1, we can conclude AHKA-1 clearly exhibits heteroresistance.

### Resistome of AHR-KA-1

To help understand the mechanisms leading to heteroresistance, the AHR-KA-1 genome was sequenced. Draft genome assembly of AHR-KA-1 identified a single circular chromosome measuring 5,505,038 base pairs in length, with an average GC content of 54.82%, which is similar to other strains of *K. aerogenes*. The resistome analyses found 15 antimicrobial resistance genes, listed in Table 2. These included four β-lactamases (*bla*_OXA-1_, *bla*_CTX-M-15_, *bla*_CMY-2_, and *bla*_AmpH_), one aminoglycosides/quinolone gene (*aac(6')-Ib-cr*), one quinolone gene (*qnrB-1*), one trimethoprim gene (*dfrA14*), one fosfomycin gene (*fosA5*), one chloramphenicol gene (*catB3*), and six peptide antibiotic genes (*eptA*, *eptB*, *bacA*, *arnT*, *arnA,* and *arnC*). It also possessed a substantial number of multidrug efflux genes, as well as genes encoding outer membrane proteins. The latter, when altered through changes in expression or mutation, can contribute to antibiotic resistance against various drug classes, including aminoglycosides (*acrD*, *cpxA*, *emrE*) (Table S3).

**TABLE 2:** Identification of antibiotic resistance genes in the genome of AHR-KA-1

Phenotypic antibiotic class	Phenotypic antibiotic resistance	Reference sequence (NCBI)	Putative resistance genes	Resistance gene/protein, mechanism function	Size (aa)	Coverage	Aa identity (%)	Resistance gene characterization
Beta-lactam	Ampicillin-sulbactam, Piperacillin-tazobactam, Cefepime Cefuroxime sodium, Cefuroxime axetil, Cefoxitin, Ceftazidime, Ceftriaxone Cephamycin Penicillin G, Cefoxitin, Cephalosporin C	WP_232551847.1	*bla*_OXA-1_	Class D β-lactamase OXA	276	100	99.64	CARD, ResFinder, Arg-Annot, KEGG, Bakta
		WP_000239590.1	*bla*_CTX-M-15_	Class A extended-spectrum β-lactamases CTX-M	291	100	100	CARD, ResFinder, Arg-Annot, KEGG, Bakta
		WP_063406924.1	*bla_CMY-2_*	Class C plasmid-mediated beta-lactamase CMY	381	98	100	CARD, Arg-Annot, KEGG, Bakta
		WP_015368035.1	*bla*_AmpH_	Class C extended-spectrum β-lactamases AmpH	386	99	99.74	Card, Arg-Annot, KEGG, Bakta
Aminoglycosides/ quinolone	Amikacin, gentamicin	WP_001749987.1	*aac(6')*-Ib-cr (variant of the aacA4 gene)	Fluoroquinolone-acetylating aminoglycoside 6′ N-acetyltransferase	199	100	100	CARD, ResFinder, Arg-Annot, KEGG, Bakta
Quinolones	Ciprofloxacin	WP_014386481.1	*qnrB*1	Plasmid-mediated quinolone resistance	214	100	100	CARD, ResFinder, Arg-Annot, KEGG, Bakta
Trimethoprim	Trimethoprim	WP_004201280.1	*dfrA14*	Trimethoprim-resistant dihydrofolate reductase dfrA	157	100	100	CARD, ResFinder, Arg-Annot, KEGG, Bakta
Fosfomycin	Fosfomycin	WP_015368459.1	*fos*A5	Glutathione S-transferase	139	100	100	CARD, ResFinder, Arg-Annot, KEGG, Bakta
Phenicol	Chloramphenicol	WP_012783949.1	*catB3*	Chloramphenicol acetyltransferase	182	100	100	CARD, ResFinder, Arg-Annot, KEGG, Bakta
Peptide antibiotic	Polymyxin Bacitracin	WP_015367609.1	*ept*A	Phosphoethanolamine transferase	547	100	100	Card, KEGG, Bakta, BLAST
		WP_015369228.1	*ept*B	Phosphoethanolamine transferase	563	100	100	Card, KEGG, Bakta, BLAST
		WP_015369287.1	*arn*T	Phosphoethanolamine transferase	551	100	100	Card, KEGG, Bakta, BLAST
		WP_015369285.1	*arnA*	Phosphoethanolamine transferase	661	100	100	Card, KEGG, Bakta, BLAST
		WP_015369284.1	*arnC*	Phosphoethanolamine transferase	327	100	100	Card, KEGG, Bakta, BLAST
		WP_015369634.1	*bacA*	Undecaprenyl-diphosphate phosphatase	273	87	100	Card, KEGG, Bakta, BLAST

**Fig 4:**
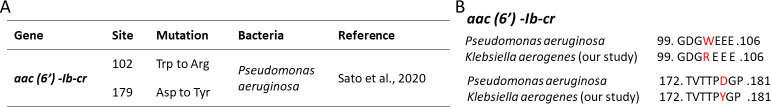
Mutations linked to amikacin resistance were detected in the AHR-KA-1 isolate. (**A**) Known resistance-conferring mutations in *aac(6’)-Ib-cr*. (**B**) Alignment of the amino acid substitutions, Trp102Arg, and Asp179Tyr, linked with amikacin resistance of *Pseudomonas aeruginosa* and AHR-KA-1, i*.*e*.*, *K. aerogenes* (our study).

The *aac(6’)-Ib-cr* gene present in AHR-KA-1 carried amino acid substitutions at positions 102 (Trp102Arg) and 179 (Asp179Tyr) (Fig. 4A and B), both of which are also associated with amikacin heteroresistance (49). Resistance to amikacin may also arise from mutations in *cpxA* (50, 51) and the *cpxA* gene in AHR-KA-1 carried 24 point mutations previously occurring in *Escherichia coli* (NCBI Ref. Seq., NC_000913.3) and 20 in *Salmonella enterica* (NCBI Ref. Seq., NC_003197.2). However, notably, none of these mutations are documented to confer amikacin resistance.

### Aminoglycoside resistance genes are highly expressed in AHR-KA-1

Stemming from these findings, we investigated whether aminoglycoside resistance genes are differentially expressed in the heteroresistant isolate AHR-KA-1 before and after drug-free passaging. We focused on four characterized genes: *aac(6′)-Ib-cr*, which encodes an aminoglycoside-modifying enzyme that inactivates drugs such as amikacin (52, 53); *acrD*, an RND-family efflux pump that exports aminoglycosides (54); *cpxA*, a component of the CpxAR two-component system that regulates envelope stress responses and can indirectly enhance aminoglycoside tolerance by modulating membrane integrity, porin expression, and efflux activity (55); and *emrE*, a small SMR-family multidrug transporter that may contribute to intrinsic aminoglycoside tolerance in certain bacteria (56, 57). Quantitative reverse transcription-PCR was used to quantify gene expression, enabling detection of up- or downregulation that may contribute to heteroresistance, which is often undetected by standard susceptibility testing (58, 59).

**Fig 5:**
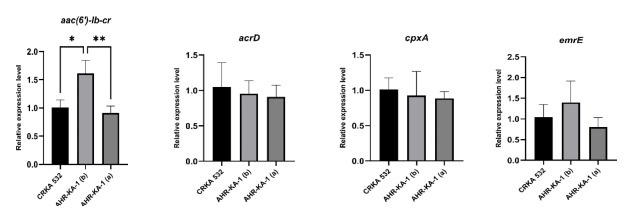
Aminoglycoside resistance gene expression levels. Elevated expression of the *aac(6’)-Ib-cr* gene was observed in AHR-KA-1, but only before drug-free passaging. CRKA 532 was included as the parental control to determine whether elevated *aac(6’)-Ib-cr* expression was specific to the heteroresistant isolates. The cultures were grown with the antibiotic (b) or after 10 days of drug-free growth (a). Statistical significance (Kruskal-Wallis): *P* < 0.05 (*), *P* < 0.01 (**).

Comparative analyses against the parental strain revealed that *aac(6′)-Ib-cr* was the only gene exhibiting significantly elevated expression in AHR-KA-1, and this increase was observed only in cultures prior to drug-free passaging (Fig. 5).

## DISCUSSION

Heteroresistance is a concerning phenomenon that can lead to failures in antibiotic treatment, thereby accelerating the development of antibiotic-resistant bacteria (60). However, the genetic mechanisms underlying the emergence and persistence of the heteroresistance phenotype remain poorly understood (61). In this study, we identified heteroresistance to amikacin in *K. aerogenes* isolates and performed an in-depth genomic analysis of one *K. aerogenes* isolate to gain a more comprehensive understanding of its genomic diversity and resistance mechanisms.

Studies have shown that heteroresistance is typically characterized by the presence of one or more subpopulations occurring at a frequency greater than 10^−7^ with resistance levels at least eightfold higher than the primary susceptible population’s breakpoint (24, 62). Consistent with this definition, five subpopulations of *K. aerogenes* resistant to amikacin were determined to be heteroresistant, as measured by the PAP assay, the gold standard for evaluating the size and resistance level of heteroresistant subpopulations (63, 64). When evaluating their stabilities, however, only one isolate exhibited a stable heteroresistant phenotype, demonstrating that the resistant subpopulations can be distinct and affirming previous studies that reported similar findings in carbapenem-resistant *K. pneumoniae* isolates (11), *Acinetobacter baumannii* (20), and pan-resistant *K. pneumoniae* isolates (65). However, to the best of our knowledge, this is the first report of amikacin heteroresistance in clinical *K. aerogenes* isolates in Brazil.

Next, biochemical tests were performed to assess the phenotypic characteristics of all five AHR-KA isolates. The indistinguishable biochemical profiles among these isolates indicated phenotypic homogeneity, a feature observed among clonal populations within Enterobacterales, including *Klebsiella* spp., particularly in nosocomial and outbreak-associated settings (66, 67). Such phenotypic uniformity is commonly used as preliminary evidence of clonality prior to confirmation by molecular or genomic typing methods (3, 68, 69). Additionally, the biochemical profiles observed for the AHR-KA isolates were characterized by positive ODC activities and the absence of malonate utilization, results that are consistent with the classical phenotypic description of *K. aerogenes* and support the initial species-level assignment based on conventional biochemical testing (46, 47). However, due to phenotypic overlap among *Klebsiella* spp. and other Enterobacterales, definitive taxonomic assignment requires confirmation by molecular or genomic approaches, which offer higher discriminatory power (47, 66, 69), and was performed as discussed below.

In the present study, the stable AHR-KA-1 strain exhibited a slightly lower growth rate compared to the parental and unstable AHR-KA-2 to 5 strains following the removal of amikacin. The current findings are consistent with the studies conducted by Zhang et al. (11), which indicated that reduced growth rates are not the primary mechanisms driving amikacin heteroresistance in stable amikacin heteroresistant *K. pneumoniae*. Similarly, AHR-KA-1 biofilms were less robust than the other strains, particularly after antibiotic-free growth. Biofilms are complex communities of bacteria surrounded by extracellular polymers and may impede the penetration of antibiotics (12, 70). At present, the impact of bacterial biofilms on heteroresistance remains poorly understood, despite their known heterogeneous response to antibiotic treatment (71). While our study revealed that one unstable heteroresistant strain exhibited increased biofilm formation after antibiotic-free passaging, the stable AHR-KA-1 strain demonstrated weak biofilm formation both before and after passages in the absence of amikacin. Consistent with the findings of Zhang et al. (11), these results suggest that biofilm formation is not the underlying mechanism driving amikacin heteroresistance. However, given that the loss of heteroresistance was associated with increased biofilm formation in two strains, further studies are required to precisely understand the relationship between these two phenotypes.

Carbapenemases are enzymes that can be encoded by chromosomal or plasmid-borne genes (72). These are considered the most potent β-lactamases, capable of hydrolyzing a wide range of β-lactam antibiotics, including carbapenems, cephalosporins, penicillins, and aztreonam (72). In our study, all amikacin heteroresistant *K. aerogenes* isolates carried two genes encoding these enzymes, i.e*.*, *bla*_KPC_ and *bla*_NDM_. However, several of the heteroresistant clones, including the stable AHR-KA-1, lost one or both of these genes. We hypothesized that while some plasmid-encoded resistance mechanisms may not incur a significant fitness cost, other plasmids carrying antibiotic resistance genes do impose a fitness burden on the host bacterium in the absence of antibiotics, enhancing the loss of plasmids (73, 74).

We performed time-kill kinetics on the stable heteroresistant AHR-KA-1 isolate, both before and after removing amikacin. We found that while the initial amikacin monotherapy could reduce the number of resistant cells in the culture media, regrowth occurred quickly, within 4–8 h. This indicated that the regrowth was a direct result of the continued proliferation of this resistant subpopulation during the amikacin exposure (75). Such regrowth dynamics are a characteristic of heteroresistance and have been reported across Gram-negative pathogens exposed to aminoglycosides and other antibiotic classes (15, 20, 76). Consistent with previous studies, these findings suggest that heteroresistance can compromise antimicrobial efficacies and lead to treatment failure, even in isolates classified as susceptible by standard susceptibility testing and when resistant subpopulations are present at very low frequencies (19, 65).

Studies have demonstrated that stable heteroresistance, which is maintained in the absence of antibiotic pressure, can be attributed to point mutations, small deletions, or insertion sequences (19, 77), and has been identified in carbapenem-resistant *A. baumannii* (75), carbapenem-resistant *K. pneumoniae* (11), and *K. pneumoniae* (77). Therefore, whole-genome sequencing analysis was conducted on the amikacin-heteroresistant isolate AHR-KA-1 to elucidate the mechanisms underlying its stable heteroresistant phenotype. The analysis of the draft genome sequence of the AHR-KA-1 isolate revealed the presence of a single circular chromosome with a length analogous to that of most *K. aerogenes* genomes deposited in the NCBI GenBank database. This confirmed the species assignment suggested by initial biochemical tests. The genome was found to harbor genes conferring resistance to different antibiotic classes, including β-lactams (*bla*_ampH_, *bla*_CTX-M-15_, *bla*_OXA-1_), trimethoprim (*dfrA14*) (78), chloramphenicol (*catB3*) (79), peptide antibiotics (*eptA*, *eptB, bacA, arnT, arnA, arnC)* (22, 80, 81), and fosfomycin (*fosA5*). This highlights the extensive resistome carried by this strain and reflects its MDR potential, which has been increasingly recognized in *K. aerogenes* clinical isolates worldwide (22, 82). One important finding was the discovery that the *aac(6’)-Ib-cr* gene in our isolate carries the D179Y and R102W mutations, which extends its resistance profile to include amikacin in *K. pneumoniae* (83) and *E. coli* (84). The *aac(6’)-Ib-cr* variant encodes a bifunctional acetyltransferase, capable of acetylating and inactivating both aminoglycoside and quinolone antibiotics (85). Consequently, we evaluated the expression levels of genes associated with aminoglycoside resistance present within AHR-KA-1. Our analysis demonstrated a statistically significant increase in the expression of *aac(6’)-Ib-cr* prior to drug-free passaging compared to the parental CRKA532 strain. The expression of this gene significantly decreased after a drug-free passage, however. Given the stability of AHR-KA-1 resistance to amikacin, therefore, the mutated *aac(6’)-Ib-cr* variant gene appears sufficient for this phenotype to be maintained.

While the findings in this study provide important information related to heteroresistance in *K. aerogenes*, it has limitations that warrant acknowledgment. The absence of whole-genome sequencing data for the unstable heteroresistant isolates prevented direct genomic comparisons with the stable isolate and the parental strain, limiting identification of the genetic determinants responsible for the observed phenotypic differences. Additionally, the small sample size, one stable and four unstable AHR-KA isolates, may limit the generalizability of the findings and the detection of rarer heteroresistant phenotypes. Finally, without clinical outcome data, we cannot directly evaluate the impact of aminoglycoside heteroresistance on treatment efficacy or patient prognosis in *K. aerogenes* infections.

Despite these limitations, our findings demonstrate that heteroresistance in *K. aerogenes* is both a complex and clinically relevant phenomenon. We observed amikacin heteroresistance in carbapenemase-producing isolates, including one strain exhibiting a stable, mutation-associated phenotype. The low fitness cost of this stable isolate, coupled with its ability to regrow under high amikacin pressure, underscores the potential impact of heteroresistant subpopulations on treatment outcomes. Comprehensive genomic analysis of this stable amikacin-resistant isolate provided valuable insights into the genetic mechanisms driving resistance. Collectively, these data highlight the critical need for detecting heteroresistant subpopulations in clinical settings, emphasize the importance of enhanced surveillance to prevent therapeutic failures, and underscore the necessity of advancing susceptibility testing methods to accurately identify heteroresistant strains.

## Data Availability

The draft genome is available at GenBank BioSample accession SAMN44283669.

## AFTER EPUB

*[This article was published on 26 February 2026 with an incorrect grant number in Funding. The error was corrected in the current version, posted on 12 March 2026.]*

## SUPPLEMENTAL MATERIAL

Supplemental tables spectrum.02833-25-s0001.docx Tables S1 to S3.

ASM does not own the copyrights to Supplemental Material that may be linked to, or accessed through, an article. The authors have granted ASM a non-exclusive, world-wide license to publish the Supplemental Material files. Please contact the corresponding author directly for reuse.
