# Supplementary material for: Heteroresistance to amikacin in Klebsiella aerogenes isolates from patients in an intensive care unit in Brazil
Source: Microbiol Spectr. 2026 Feb 26;14(4):e02833-25. doi: 10.1128/spectrum.02833-25 (PMC13055279; doi:10.1128/spectrum.02833-25)
Supplement: Supplemental tables — Tables S1 to S3. [file spectrum.02833-25-s0001.docx]

**Table S1.** Sequences of primers used for detection of resistance markers.

| **Resistance markers** | **Gene** | **Sequence (5′-3′), F/R** | **Amplicon size (bp)** | **Reference** |
| --- | --- | --- | --- | --- |
| Beta-Lactam | *bla*_TEM_ | TGCGGTATTATCCCGTGTTG  TCGTCGTTTGGTATGGCTTC | 296 | Xiong et al., 2007 |
|  | *bla*_CTX-M-1 group (CTX-M-1,-3,-10,-11 and -12)_ | ACAGCGATAACGTGGCGATG  TCACCCAATGCTTTACCCAG | 216 | Xiong et al., 2003 |
|  | *bla*_SHV variants_ | AGCCGCTTGAGCAAATTAAAC  ATCCCGCAGATAAATCACCAC | 712 | Dallenne et al, 2010 |
|  | *bla*_KPC_ | CGTCTAGTTCTGCTGTCTTG  CTTGTCATCCTTGTTAGGCG | 797 | Poirel et al., 2011 |
|  | *bla*_NDM_ | GCAGCTTGTCGGCCATGCGGGC  GGTCGCGAAGCTGAGCACCGCAT | 782 | Doyle et al.; 2012 |
| Aminoglycosides | *arm*A | ATTCTGCCTATCCTAATTGG  ACCTATACTTTATCGTCGTC | 315 | Sheikhalizadeh et al., 2017 |
|  | *rmt*B | ATGAACATCAACGATGCCCT  CCTTCTGATTGGCTTATCCA | 769 | Yan et al., 2004 |
|  | *aph (3′)*-VIa *(aph*A6) | CGGAAACAGCGTTTTAGA  TTCCTTTTGTCAGGTC | 716 | Noppe-Leclerq et al., 1999 |
|  | *aac-(6’)-Ib* | TATGAGTGGCTAAATCGAT  CCCGCTTTCTCGTAGCA | 395 | Huang Jinwei et al., 2012 |
| Aminoglycosides/Quinolone | *aac (6′)-Ib-cr* | ATGACTGAGCATGACCTTGC  TTAGGCATCACTGCGTGTTC | 519 | Platell et al., 2011 |
| **Resistance markers used in qRT-PCR analysis** | **Gene** | **Sequence (5′-3′), F/R** | **Amplicon size (bp)** | **Reference** |
| Aminoglycosides/Quinolone | *aac (6’)-Ib-cr* | TGACCTTGCGATGCTCTATG  TGCAATGTATGGAGTGACGG | 148 | This study |
| Efflux pump | *acrD* | TTACCGCCAGTTCTCCATTAC  TGACCATGTACTTCGCCTTTG | 126 | This study |
|  | *emrE* | CTCTCAGGTCGTTAAGGTCATG  CCGACAGTATCAAGCCCATC | 149 | This study |
| Efflux pump regulator | *cpxA* | TGGACAGCATGATTAACGACC  AAGGATTTACCCATCTGCTCG | 145 | This study |
| Gene housekeeping | *16S rRNA* | AGGCTAGAGTCTTGTAGAGGG  AGCGTCAGTCTTTGTCCAG | 110 | This study |

**Table S2.** Identification of multidrug efflux systems, including their associated activators and regulators, as well as the outer membrane proteins, within the genome of the AHR-KA-1 strain.

| **Type** | **Antibiotic Resistance** | **Reference Sequence (NCBI)** | **Putative resistance genes** | **Resistance gene/protein, mechanism function** | **Size (aa)** | **Coverage**  **(%)** | **aa identity (%)** | **Resistance Gene Characterization** |
| --- | --- | --- | --- | --- | --- | --- | --- | --- |
| **Multidrug Efflux** | Quinolones, Tigecycline | WP_015367128.1 | *oqx*A | Multidrug efflux RND transporter periplasmic adaptor subunit A | 391 | 100 | 84.35 | CARD, Arg-Annot, KEGG, Bakta |
|  |  | WP_015367127.1 | *oqx*B | Multidrug efflux RND transporter periplasmic adaptor subunit B | 1050 | 100 | 100 | CARD, Arg-Annot, KEGG, Bakta |
|  | Tetracycline, Glycylcycline, Penam, Cephalosporin, Phenicol, Rifamycin, Fluoroquinolone, Tigecycline, Disinfecting Agents and Antiseptics | WP_020079451.1 | *acr*A | Multidrug efflux pump subunit A | 399 | 100 | 100 | CARD, KEGG, Bakta, BLAST |
|  |  | WP_015367916.1 | *acr*B | Multidrug efflux pump subunit B | 1048 | 100 | 100 | CARD, KEGG, Bakta, BLAST |
|  | Aminoglycosides | WP_047080751.1 | *acrD* | Multidrug efflux pump subunit D | 1037 | 100 | 100 | CARD, KEGG, Bakta, BLAST |
|  | Cephamycin, Cephalosporin, Fluoroquinolone | WP_015369449.1 | *acrE* | Multidrug efflux pump subunit E | 379 | 100 | 100 | CARD, BLAST |
|  | Cephalosporin, Fluoroquinolone | WP_015369448.1 | *acrF* | Efflux RND transporter permease subunit AcrF | 1036 | 100 | 100 | CARD, BLAST |
|  | Phosphonic acid antibiotic | WP_015367213.1 | *mdtG* | Multidrug efflux MFS transporter MdtG | 416 | 100 | 100 | CARD, BLAST, Bakta |
|  | Fluoroquinolone, Disinfecting Agents and Antiseptics, Phenicol | WP_015368446.1 | *mdtM* | Multidrug efflux MFS transporter MdtM | 414 | 100 | 100 | CARD, KEGG, Bakta, BLAST |
|  | Chloramphenicol, Norfloxacin, Acriflavine | WP_015367543.1 | *kde*A | MdfA family multidrug efflux MFS transporter | 410 | 100 | 100 | BLAST, Bakta |
|  | Aminocoumarin | WP_163365960.1 | *mdtC* | Multidrug efflux MFS transporter | 1025 | 100 | 99.9 | CARD, KEGG, Bakta, BLAST |
|  | Aminocoumarin | WP_015365786.1 | *mdt*A | MuxA family multidrug efflux RND transporter periplasmic adaptor subunit | 414 | 100 | 100 | CARD, KEGG, Bakta, BLAST |
|  | Aminocoumarin | WP_149270490.1 | *mdt*B | MuxB family multidrug efflux RND transporter periplasmic adaptor subunit | 1040 | 100 | 99.90 | CARD, KEGG, Bakta, BLAST |
|  | Nucleoside antibiotic | WP_032706346.1 | *mdtN* | Multidrug transporter subunit MdtN | 342 | 100 | 100 | CARD, KEGG, Bakta, BLAST |
|  | Fluoroquinolone | WP_015366890.1 | *mdt*K | MdtK family multidrug efflux MATE transporter | 457 | 100 | 100 | KEGG, BLAST, Bakta |
|  | Peptide antibiotic, Tetracycline, Aminoglycosides, Cephalosporin, Phenicol, Rifamycin, Fluoroquinolone, Disinfecting Agents and Antiseptics | WP_015369648.1 | *tolC* | Outer membrane channel protein TolC | 484 | 100 | 100 | CARD, KEGG, BLAST, Bakta |
|  | Peptide antibiotic | WP_015365632.1 | *yojI* | Multidrug ABC transporter permeasse/ATP binding protein | 547 | 100 | 100 | CARD |
|  | Nitroimidazole antibiotic | WP_015367468.1 | *msbA* | Lipid A ABC transporter ATP binding protein/permeasse MsbA | 582 | 100 | 100 | CARD, KEGG, Bakta, BLAST |
|  | Peptide antibiotic, Aminoglycoside, Cephalosporin, Carbapenem, Fluoroquinolone, Macrolide | WP_015370068.1 | *emrA* | MFS transporter periplasmic adaptor subunit EmrA | 390 | 100 | 100 | Bakta, BLAST |
|  | Fluoroquinolone | WP_020078123.1 | *emrB* | MFS transporter permease subunit EmrA | 512 | 100 | 100 | Bakta, BLAST |
|  | Aminoglycosides, Macrolide | WP_015367055.1 | *emrE* | SMR Family transporter emrE | 111 | 100 | 100 | CARD, KEGG, Bakta, BLAST |
|  | Fluoroquinolone | WP_015366270.1 | *norB* | MFS transporter NorB | 463 | 100 | 100 | CARD, BLAST |
|  | Disinfecting Agents and Antiseptics, Macrolide | WP_015366451.1 | *amvA* | MFS transporter AmvA | 496 | 100 | 100 | CARD |
|  | Rifamycin, Aminocoumarin, Peptide antibiotics  Tetracyclines | WP_020079626.1 | *lptD* | LPS-assembly protein LptD | 782 | 98 | 100 | CARD, Bakta, BLAST |
|  |  | WP_000804064.1 | *tet(A)* | Tetracycline efflux MFS transporter Tet(A) | 399 | 100 | 100 | CARD, ResFinder, Arg-Annot, BLAST, Bakta |
| **Multidrug Efflux Activators and Regulators** | Tetracycline, Cephalosporin, Phenicol, Glycylcycline, Penam, Fluoroquinolone, Rifamycin, Cephamycin, Disinfecting agents and Antiseptics | WP_015368733.1 | *soxR* | Redox-sensitive transcriptional activator SoxR | 152 | 100 | 100 | CARD, KEGG, BLAST, Bakta |
|  | Fluoroquinolone, Macrolide | WP_305210200.1 | *crp* | cAMP-activated global transcriptional regulator | 215 | 100 | 100 | CARD, KEGG, Bakta, BLAST |
|  | Tetracycline, Disinfecting Agents and Antiseptics, Phenicol, Riphamycin | WP_196092912.1 | *sdiA* | Transcriptional regulator SdiA | 230 | 100 | 100 | CARD, KEGG, Bakta, BLAST |
|  | Phenicol Antibiotic, Diaminopyrimidine Antibiotic, Fluoroquinolone Antibiotic | WP_000906486.1 | *rsmA* | Carbon storage regulator | 61 | 100 | 100 | CARD, KEGG, BLAST, Bakta |
|  | Tetracycline, Fluoroquinolone, Macrolide | WP_004103116.1 | *h-ns* | Histone-like nucleoid-structuring protein H-NS | 135 | 100 | 100 | CARD, KEGG, Bakta, BLAST |
|  | Amikacin and other Aminoglycosides, Aminoumarin | WP_015368938.1 | *cpxA* | Envelope stress sensor histidine kinase cpxA | 457 | 100 | 100 | CARD, KEGG, Bakta, BLAST |
|  | Aminoglycosides | WP_015367692.1 | *kdpE* | Two-component system response regulator kdpE | 225 | 100 | 100 | CARD, KEGG, Bakta, BLAST |
|  | Fluoroquinolone | WP_015370069.1 | *MprA* | Multidrug efflux transporter EmrAB transcrptional repressor | 176 | 100 | 100 | CARD, KEGG, Bakta, BLAST |
|  | Tetracycline, Disinfecting Agents and Antiseptics, Phenicol, Riphamycin | WP_045362589.1 | *acrS* | AcrEF operon transcriptional regulator | 218 | 90 | 99.54 | CARD, KEGG, Bakta, BLAST |
|  | Fluoroquinolone, Carbapenem, Cephalosporin, Glycylcycline, Disinfecting Agents and Antiseptics | WP_015367806.1 | *ramA* | RamA Family antibiotic efflux transcriptional regulator | 113 | 100 | 100 | CARD, BLAST |
|  | Nucleoside antibiotics, Disinfecting Agents and Antiseptics | WP_015368259.1 | *leuO* | Transcriptional regulator LeuO | 322 | 100 | 100 | CARD, BLAST |
|  | Fluoroquinolone, Carbapenem, Cephalosporin, Glycylcycline | WP_015366733.1 | *marA* | MDR efflux pump AcrAB transcriptional activator MarA | 127 | 100 | 100 | CARD, BLAST |
|  | Cyprofloxacin, Tetracycline | WP_015366732.1 | *mar*R | Multiple antibiotic resistance transcriptional regulator | 144 | 100 | 100 | KEGG, Bakta, BLAST |
| **Outer Membrane Proteins** | Carbapenems, Cephalosporin,  Monobactam | WP_015365731.1 | *mdtQ* | Outer membrane protein MdtQ | 450 | 100 | 99.33 | Bakta, BLAST |
|  | Cephalosporin, Carbapenem, Penam, Monobactam, Cephamycin | WP_015365887.1 | *omp*C | Outer membrane protein OmpC | 380 | 100 | 100 | Bakta,BLAST |
|  |  | WP_015367884.1 | *ompC* | Porin OmpC | 381 | 100 | 100 | Bakta,BLAST |
|  |  | WP_015365628.1 | *ompC* | Porin OmpC | 375 | 100 | 100 | Bakta,BLAST |
|  | Peptide Antibiotic/Beta-lactam | WP_015366093.1 | *omp*A_C-*like* | Peptidoglycan binding domains similar to the C-terminal domain of outer-membrane protein OmpA | 560 | 100 | 100 | BLAST |
|  |  | WP_080473199.1 | *omp*A | Outer membrane protein A | 350 | 100 | 100 | CARD, BLAST, Bakta |
|  |  | WP_015367453.1 | *ompk35* |  | 359 | 100 | 100 | Bakta, BLAST |
|  |  | WP_015367577.1 | *omp*X | Outer membrane protein X | 171 | 100 | 100 | Bakta, BLAST |
|  |  | WP_032706228.1 | *omp*W | Outer membrane protein W | 212 | 100 | 100 | Bakta, BLAST |

**Table S3**. Biochemical characteristics of amikacin-heteroresistant *K. aerogenes* isolates.

| Test | AHR-KA-1 (b) | AHR-KA-1 (a) | AHR-KA-2 (b) | AHR-KA-2 (a) | AHR-KA-3 (b) | AHR-KA-3 (a) | AHR-KA-4 (b) | AHR-KA-4 (a) | AHR-KA-5 (b) | AHR-KA-5 (a) |
| --- | --- | --- | --- | --- | --- | --- | --- | --- | --- | --- |
| Ortho-nitrophenyl-β-galactopyranoside | + | + | + | + | + | + | + | + | + | + |
| Lysine | + | + | + | + | + | + | + | + | + | + |
| Ornithine | + | + | + | + | + | + | + | + | + | + |
| Sodium thiosulfate | - | - | - | - | - | - | - | - | - | - |
| Voges-Proskauer | + | + | + | + | + | + | + | + | + | + |
| Phenylalanine | - | - | - | - | - | - | - | - | - | - |
| Malonate | - | - | - | - | - | - | - | - | - | - |
| Rhamnose | + | + | + | + | + | + | + | + | + | + |
| Adonitol | + | + | + | + | + | + | + | + | + | + |
| Arabinose | + | + | + | + | + | + | + | + | + | + |
| Salicin | + | + | + | + | + | + | + | + | + | + |
| Inositol | + | + | + | + | + | + | + | + | + | + |
| Sorbitol | + | + | + | + | + | + | + | + | + | + |
| Sucrose | + | + | + | + | + | + | + | + | + | + |
| Mannitol | + | + | + | + | + | + | + | + | + | + |
| Raffinose | + | + | + | + | + | + | + | + | + | + |
| Cetrimide | + | + | + | + | + | + | + | + | + | + |
| Acetamide | - | - | - | - | - | - | - | - | - | - |
| Citrate | - | - | - | - | - | - | - | - | - | - |
| Maltose | - | - | - | - | - | - | - | - | - | - |
| Esculin | + | + | + | + | + | + | + | + | + | + |
| Arginine | - | - | - | - | - | - | - | - | - | - |
| Urea | - | - | - | - | - | - | - | - | - | - |
| Tryptophan | - | - | - | - | - | - | - | - | - | - |
